# Supplementary material for: GraphVelo allows for accurate inference of multimodal omics velocities and molecular mechanisms for single cells
Source: Res Sq. 2025 Jan 15:rs.3.rs-5613372. Preprint. [Version 1] doi: 10.21203/rs.3.rs-5613372/v1 (PMC11774466; doi:10.21203/rs.3.rs-5613372/v1)
Supplement: Supplement 1 [file NIHPPRS5613372v1-supplement-1.pdf]

Supplementary Files

This is a list of supplementary files associated with this preprint. Click to download.

- supplemental.pdf
